# Role of Color Doppler Flowmetry in Prediction of Intrauterine Growth Retardation in High-Risk Pregnancy

**DOI:** 10.7759/cureus.1827

**Published:** 2017-11-08

**Authors:** Sachin Khanduri, Saakshi Chhabra, Santosh Yadav, Tushar Sabharwal, Mriganki Chaudhary, Tarim Usmani, Aakshit Goyal, Hritik Sharma

**Affiliations:** 1 Radiodiagnosis, Era's Lucknow Medical College and Hospital

**Keywords:** iugr, color doppler flowmetry, umbilical artery pi, early third trimester, late third trimester

## Abstract

Objective: To evaluate the usefulness of Color Doppler flowmetry in the prediction of intrauterine growth restriction (IUGR) in high-risk pregnancies.

Materials and method: A total of 62 high-risk pregnant women underwent Color Doppler flowmetric umbilical artery pulsatility index (PI), resistive index (RI) and systolic/diastolic (S/D) ratio, middle cerebral artery PI, RI and S/D ratio, Ductus venosus S-wave/isovolumetric A-wave index (SIA) and vertebral artery RI at 23-27 weeks, 28-32 weeks and 32-36 weeks of their pregnancy. Cerebral-umbilical C/U PI, RI and S/D were evaluated at the third visit. All the pregnancies were followed up till delivery. Ponderal index <10 was considered to be indicative of IUGR. Data were analyzed using IBM Statistical Package for Social Sciences (SPSS) 21.0.

Results: Thirty-nine (62.9%) deliveries were IUGR. On all the three visits, umbilical artery, mean PI, RI and SD values were significantly higher while MCA PI, RI and SD values were significantly lower in IUGR as compared to non-IUGR cases. Third visit C/U PI, RI and SD ratio values were also significantly lower in IUGR as compared to non-IUGR cases. Ductus venosus SIA values did not show a significant difference between IUGR and non-IUGR groups. The vertebral artery resistive index was significantly higher in non-IUGR as compared to IUGR on all the visits. Umbilical artery PI was the most sensitive and specific for the prediction of IUGR at all the three visits, with the maximum sensitivity and specificity at the third visit (82.1% and 87%). Third visit C/U PI was most sensitive (82.1%) and specific (96.7%) for the prediction of IUGR.

Conclusion: This showed that Doppler flowmetry is a useful method for the prediction of IUGR in high-risk pregnancies.

## Introduction

Intrauterine growth restriction (IUGR) is an indicator of the increased risk of perinatal and long-term mortality and morbidity when compared to those born with normal growth. There is a considerable difference in the incidence of IUGR across different populations. In babies born with a birth weight less than 2500 gms, its prevalence is almost 33%. The incidence of IUGR shows a dependence on economic growth too, with a relatively lower incidence in developed countries (4-8%) as compared to that in developing countries (6%-30%) [1]. The average incidence of IUGR is nearly 8% in the general population. In nearly 35%-40% of the cases, IUGR is the consequence of an abnormal condition. Factors like placental insufficiency, maternal hypertension, cardiovascular disease, diabetes, infections, low socioeconomic status, previous history and preeclampsia are some of the known risk factors for IUGR [2]. Poor pregnancy outcome has shown a strong link with IUGR; more than half the stillbirths are associated with IUGR and nearly 10% of perinatal mortality is consequent to undetected IUGR [3].

For the fetus, the placenta is the only nutritional support available. During IUGR, the ability of the placenta to provide adequate nutrition to the fetus is restricted, thus resulting in developmental problems [1,4].

The maintenance of good utero-placental circulation is necessary to continue a normal pregnancy. The progression of pregnancy is marked by a number of changes and adaptations in the maternal, placental and fetal vasculatures [5]. An inability to adapt to these changes results in the development of abnormal vascular resistance patterns, which might consecutively lead to the compromise of fetal well-being and ultimately IUGR [6]. Early identification and prediction of IUGR, to a great extent, rests in an ability to evaluate the maternal, placental and fetal vascular patterns effectively and efficiently.

A number of indices based on Color Doppler flowmetry have been proposed to evaluate the risk of intrauterine growth restriction in an ongoing pregnancy – some of these include the pulsatility and resistive indices (PI and RI) of the umbilical artery (UA) and that of the middle cerebral artery (MCA), the resistive index of the fetal vertebral artery (VA) and the S-wave/isovolumetric A-wave (SIA) index of ductus venosus (DV) in predicting fetal growth restriction. In the present study, an attempt has been made to evaluate the efficacy of these Doppler indices in our settings in late second and early and late third trimester pregnancies [4,7-11].

## Materials and methods

The present study was carried out at the Department of Radiodiagnosis, Era’s Lucknow Medical College, Lucknow, in collaboration with the Department of Obstetrics and Gynaecology of the same institution after obtaining approval from the Institutional Ethical Committee and getting informed consent from all the participants. A total of 100 pregnant women attending the antenatal clinic in the Department of Obstetrics and Gynaecology, with the clinical criteria of IUGR, were recruited. The clinical examination result, obstetric color Doppler study and perinatal outcome could be evaluated in 62 women only.

The clinical criteria for suspected cases of IUGR included a history of pregnancy-induced hypertension (PIH), renal disease, cardiac disease, stage third and fourth fetal chromosomal abnormalities in initial pregnancies, toxoplasmosis, other agents, rubella, cytomegalovirus and herpes simplex (TORCH) infection, advanced insulin dependent diabetes mellitus (IDDM), smokers, patients showing longitudinal lie low symphysis fundal height, being less than the period of gestation by four weeks or more. Women with a congenital malformation in the present fetus, a multifetal pregnancy and mistaken dates were excluded from the assessment.

Gestational age determination was based on the best estimate from the last menstrual history and by ultrasonography (USG) or routine fetal biometry in the first trimester or early second trimester.

All the patients were subjected to Doppler waveform analysis on the Color Doppler machine, GE Voluson P8 (GE Healthcare, Little Chalfont, United Kingdom), using a 3.5 MHz probe.

To use Doppler velocimetry, patients were first scanned in the routine fashion using B-mode. Then, the vessels of interest were confirmed by color Doppler. The Doppler signal was then obtained by placing the Doppler gate directly over the vessel of interest. The flow velocity waveforms were obtained in periods of fetal inactivity and apnea.

Doppler velocimetry was performed on the umbilical artery, the middle cerebral artery, the vertebral artery and the ductus venosus close to the transducer. Doppler velocimetry of the umbilical arteries was performed in a free-floating loop of the mid portion of the umbilical cord away from the placental and fetal cord insertion. The umbilical cord was investigated by color flow Doppler, and flow velocity waveforms were obtained from each artery. The middle cerebral artery was visualized in a plane immediately caudal to the trans-thalamic plane used to obtain the biparietal diameter (BPD) and head circumference (HC) biometric data. Using color flow Doppler, the middle cerebral artery was identified in the Sylvian fissure arising from the Circle of Willis. The Doppler sample was placed in the first third of the middle cerebral artery and a flow velocity waveform was obtained. Doppler signals were measured by measuring peak systolic and low diastolic velocities. Pulsed wave Doppler ultrasound studies were performed in the absence of fetal breathing and fetal movements. Ductus venosus Doppler waveforms were serially studied from the time the diagnosis was made until delivery. The HC was sampled soon after its origin from the umbilical vein, either in a midsagittal longitudinal plane of the fetal trunk or in an oblique transverse plane through the upper abdomen.

In addition, Doppler indices like pulsatility index (PI), resistive index (RI), systolic/diastolic (S/D) ratio and the ratio of PI and RI will be calculated with respect to the middle cerebral artery (MCA) and umbilical artery (UA); resistive index in the vertebral artery and SIA index in ductus venosus.

The cut-off values for different Doppler parameters for the prediction of IUGR were as follows:

Umbilical artery: Pulsatility Index (PI)>1.42 [12], Resistive index (RI) >0.72 [13], S/D [14].

Middle cerebral artery: PI<1.5 [12], RI<0.59 [15], S/D<4 [10].

Cerebro-umbilical artery ratios (C/U): PI<1.08 [16], RI<1 [17], S/D<111.

Vertebral artery: RI<0.8 for first visit (23-27 weeks), RI<0.78 for second visit (28-32 weeks) and RI<0.72 (33-37 weeks) [18].

Ductus venosus SIA: SIA index normal values were considered in accordance with the normal range values provided by Picconi et al. (2008) [19] that range between -1.25 and 2.07. Thus, abnormal values predictive of IUGR were at two ends, i.e., either below -1.25 or above 2.07.

Standard treatment was given to all the patients of suspected IUGR.

After delivery birth weight (immediately within six hours) was measured on an electronic weighing machine (machine’s lower limit of measurement is less than 10 gm), Apgar’s score after five minutes of birth. Baby anthropometry, i.e., length, head circumference, abdominal circumference, upper segment and lower segment ratio were measured. Ponderal index was calculated as birth weight (in gms) per length (in cm). Ponderal index of <10 indicates growth restriction.

Data analysis: Data so collected was evaluated using IBM Statistical Package for Social Sciences (SPSS), version 21.0. Independent samples ‘t’-test was used to compare the data. A ‘p’ value (probability of chance error) less than 0.05 indicated a statistically significant association.

## Results

The age of the women enrolled in the study ranged from 18 to 37 years with a mean age of 26.13±4.21 years. A total of 39/62 (62.9%) deliveries were IUGR.

**Figure 1:**
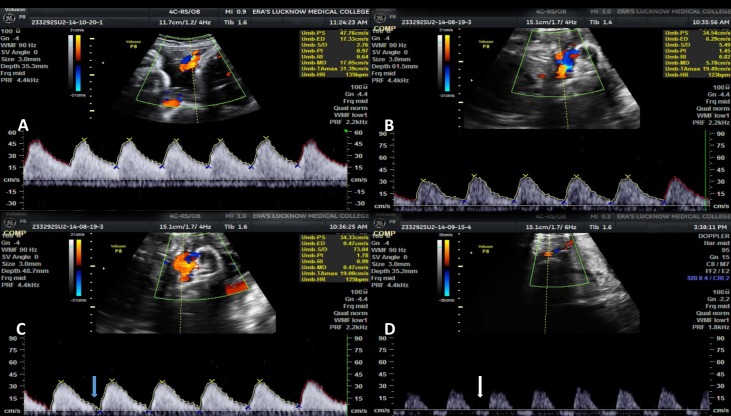
Umbilical artery waveform variations at various gestational ages A. Umbilical artery waveform at 32 weeks gestational age, showing normal diastolic flow B. Umbilical artery waveform at 30 weeks gestational age, showing reduced diastolic flow C. Umbilical artery waveform at 28 weeks gestational age, showing grossly reduced diastolic flow (blue arrow) D. Umbilical artery waveform at 35 weeks gestational age, showing reversed diastolic flow (white arrow)

**Figure 2:**
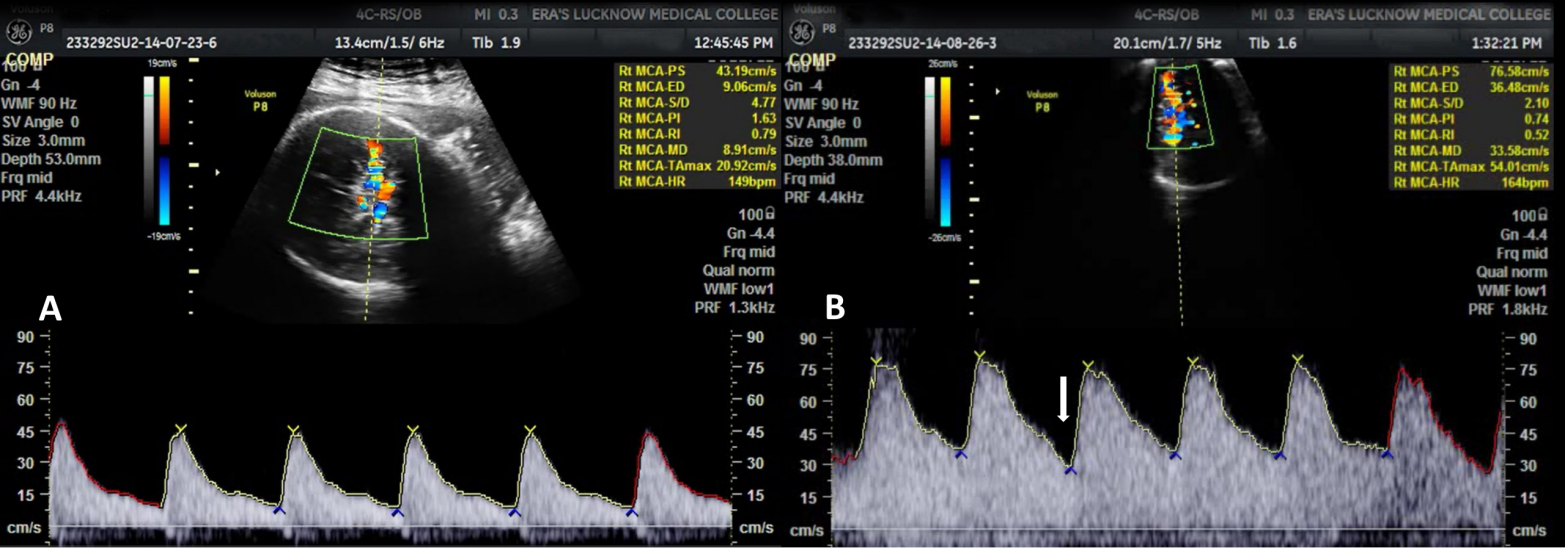
Middle cerebral artery waveform variations at various gestational ages A. Middle cerebral artery waveform at 32 weeks gestational age, showing normal flow velocity waveform B. Middle cerebral artery waveform at 33 weeks gestational age, showing increased diastolic flow (white arrow)

**Figure 3:**
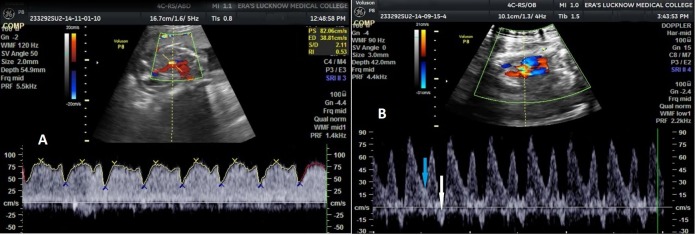
Ductus venosus waveform variations at various gestational ages A. Ductus venosus waveform at 29 weeks gestational age, showing normal flow velocity waveform B. Ductus venosus waveform at 30 weeks gestational age, showing reversal of flow (white arrow) with decreased end diastolic velocity (EDV) (blue arrow)

**Figure 4:**
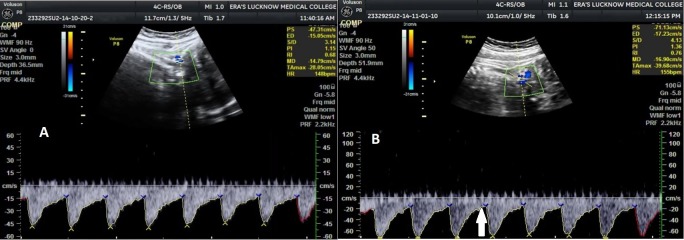
Vertebral artery waveform variations at various gestational ages A. Vertebral artery waveform at 34 weeks gestational age, showing normal flow velocity waveform B. Vertebral artery waveform at 30 weeks gestational age, showing increased diastolic flow

On all the three visits, umbilical artery, mean PI, RI and S/D values were significantly higher in IUGR as compared to non-IUGR cases (Figure 1) and MCA PI, RI and S/D values were significantly lower in IUGR as compared to non-IUGR cases (Figure 2). Third visit C/U PI, RI and S/D ratio values were also significantly lower in IUGR as compared to non-IUGR cases. Ductus venosus SIA values did not show a significant difference between IUGR and non-IUGR groups (Figure 3). The vertebral artery resistive index was significantly higher in non-IUGR as compared to IUGR on all the visits (Figure 4) (Table 1).

**Table 1:** Evaluation of different parameters at different gestational age IUGR - intrauterine growth retardation SD - standard deviation t - independent samples 't' test p - probability of chance error PI - pulsatility index RI - resistive index S/D - systolic/diastolic C/U - cerebral-umbilical SIA - S-wave/isovolumetric A-wave index

	IUGR (n=39)		No IUGR (n=23)		"t"	"p"
	Mean	SD	Mean	SD		
Umbilical artery						
PI Visit 1	1.50	0.13	1.34	0.11	4.839	<0.001
PI Visit 2	1.49	0.12	1.29	0.12	6.540	<0.001
PI Visit 3	1.49	0.12	1.23	0.13	7.708	<0.001
RI Visit 1	0.74	0.08	0.62	0.12	5.036	<0.001
RI Visit 2	0.73	0.07	0.57	0.12	6.658	<0.001
RI Visit 3	0.72	0.07	0.53	0.12	7.718	<0.001
S/D Visit 1	3.55	0.70	2.87	0.73	3.660	0.001
S/D Visit 2	3.58	0.68	2.76	0.70	4.508	<0.001
S/D Visit 3	3.69	0.67	2.62	0.70	5.982	<0.001
Middle Cerebral Artery						
PI Visit 1	1.50	0.10	1.62	0.14	-3.930	<0.001
PI Visit 2	1.54	0.11	1.69	0.14	-4.557	<0.001
PI Visit 3	1.49	0.09	1.67	0.13	-6.362	<0.001
RI Visit 1	0.61	0.10	0.70	0.09	-3.751	<0.001
RI Visit 2	0.63	0.12	0.75	0.08	-4.310	<0.001
RI Visit 3	0.61	0.12	0.72	0.09	-4.115	<0.001
S/D Visit 1	3.85	0.61	4.48	0.58	-4.016	<0.001
S/D Visit 2	3.93	0.62	4.66	0.57	-4.612	<0.001
S/D Visit 3	3.69	0.62	4.49	0.66	-4.757	<0.001
C/U Ratio at Third Visit						
PI	1.01	0.13	1.37	0.24	-7.698	<0.001
RI	0.86	0.23	1.43	0.40	-7.117	<0.001
S/D	1.04	0.30	1.91	0.84	-5.852	<0.001
Ductus Venosus SIA						
Visit 1	1.20	1.15	1.35	0.46	-0.587	0.560
Visit 2	1.18	1.20	1.43	0.46	-0.970	0.336
Visit 3	1.18	1.38	1.34	0.62	-0.530	0.598
Vertebral Artery Resistive Index						
Visit 1	0.78	0.05	0.81	0.06	-2.148	0.036
Visit 2	0.74	0.07	0.79	0.05	-3.002	0.004
Visit 3	0.68	0.06	0.75	0.04	-4.484	<0.001

At the first visit (23-27 weeks), among different modalities, umbilical artery PI was the most sensitive (61.5%) while middle cerebral artery PI was the most specific modality. Among different modalities, MCA PI also had the maximum positive predictive value. However, all the modalities had poor negative predictive values. Among different modalities, UA PI had the maximum negative predictive value. As far as accuracy was concerned, both UA PI as well as UA RI had equal diagnostic accuracy. At the second visit (28-32 wks), among different parameters evaluated, once again umbilical artery PI had maximum sensitivity while MCA RI had maximum specificity. The maximum positive predictive value was observed for umbilical artery RI and maximum negative predictive value for umbilical artery PI. As far as diagnostic accuracy was concerned, the maximum value (75.8%) was shared by UA PI and UA RI. At the third visit, umbilical artery PI and C/U PI had the maximum sensitivity (82.1%), however, C/U PI had the maximum specificity (96.7%). Positive predictive and negative predictive values were also observed to be the maximum for C/U PI. Overall diagnostic accuracy was also found to be the maximum for C/U PI (Table 2).

**Table 2:** Evaluation of diagnostic efficacy of different parameters at different gestational age IUGR - intrauterine growth retardation Sens - sensitivity Spec - specificity PPV - positive predictive value NPV - negative predictive value PI - pulsatility index RI - resistive index S/D - systolic-diastolic C/U - cerebral-umbilical SIA - S-wave/isovolumetric A-wave index

SN	Visit No.	IUGR (n=39)				Non-IUGR (n=23)			Sens	Spec	PPV	NPV	Accuracy
		+ve		-ve		+ve		-ve					
Umbilical Artery													
1.	PI Visit 1	24	15		6		17		61.5	73.9	80.0	53.1	66.1
2.	PI Visit 2	29	10		5		18		74.4	78.3	85.3	64.3	75.8
3.	PI Visit 3	32	7		3		20		82.1	87.0	91.4	74.1	83.9
4.	RI Visit 1	23	16		5		18		59.0	78.3	82.1	52.9	66.1
5.	RI Visit 2	27	12		3		20		69.2	87.0	90.0	62.5	75.8
6.	RI Visit 3	27	12		2		21		69.2	91.3	93.1	63.6	77.4
7.	S/D Visit 1	21	18		4		19		53.8	82.6	84.0	51.4	64.5
8.	S/D Visit 2	23	16		4		19		59.0	82.6	85.2	54.3	67.7
9.	S/D Visit 3	24	15		3		20		61.5	87.0	88.9	57.1	71.0
Mid Cerebral artery													
1.	PI Visit 1	16	23		3		20		41.0	87.0	84.2	46.5	58.1
2.	PI Visit 2	16	23		3		20		41.0	87.0	84.2	46.5	58.1
3.	PI Visit 3	20	19		2		21		51.3	91.3	90.9	52.5	66.1
4.	RI Visit 1	14	25		4		19		35.9	82.6	77.8	43.2	53.2
5.	RI Visit 2	16	23		2		21		41.0	91.3	88.9	47.7	59.7
6.	RI Visit 3	17	22		3		20		43.6	87.0	85.0	47.6	59.7
7.	S/D Visit 1	13	26		6		17		33.3	73.9	68.4	39.5	48.4
8.	S/D Visit 2	16	23		5		18		41.0	78.3	76.2	43.9	54.8
9.	S/D Visit 3	18	21		5		18		46.2	78.3	78.3	46.2	58.1
C/U at Third Visit													
1.	PI	32	7		1		22		82.1	95.7	97.0	75.9	87.1
2.	RI	28	11		4		19		71.8	82.6	87.5	63.3	75.8
3.	S/D	30	9		5		18		76.9	78.3	85.7	66.7	77.4
Ductus Venosus SIA													
1.	1^st^ Visit	10	29		4		19		25.6	82.6	71.4	39.6	46.8
2.	2^nd^ Visit	11	28		3		20		28.2	87.0	78.6	41.7	50.0
3.	3^rd^ Visit	16	23		2		21		41.0	91.3	88.9	47.7	59.7
Vertebral Artery RI													
1.	1^st^ Visit	17	22		11		12		43.6	52.2	60.7	35.3	46.8
2.	2^nd^ Visit	22	17		9		14		56.4	60.9	71.0	45.2	58.1
3.	3^rd^ Visit	28	11		6		17		71.8	73.9	82.4	60.7	72.6

## Discussion

The prevalence of IUGR in clinically suspect cases of fetal growth restriction was 62.9% as observed in the present study. Generally, IUGR affects 3% to 10% of normal pregnancies [20], the higher prevalence of IUGR in the present study could be attributed to the fact that the pregnancies included in the present study were already diagnosed as clinically suspect IUGR. A high prevalence of IUGR among clinically suspect IUGR is a known fact. A high prevalence of IUGR in clinically suspect cases is reported in literature too. Chanprapaph et al. (2004) [21], in their study, reported an IUGR prevalence of 50.9% using a similar study design as ours.

In the present study, the discriminant role of different parameters was assessed by primarily differentiating between the IUGR and non-IUGR groups with the help of a comparison of the mean values of different parameters. A difference in the mean values of all the parameters at all the visits, except ductus venosus SIA, was observed, thus establishing that at least these parameters have a discriminant ability. However, for ductus venosus, no significant difference between the two groups was observed. On exploring the issue further, it was found that ductus venosus SIA follows a unique pattern with two cut-off ranges – one at -1.25 and another at >2.07 (Picconi et al., 2008) [19]; this practically is a complex situation in which the evaluation of the mean value for discrimination between two adverse outcomes is not feasible.

In the present study, for umbilical artery PI>1.42, the sensitivity was 61.5%, 74.4% and 82.1%, specificity was 73.9%, 78.3% and 87.0%, positive predictive value was 80.0%, 85.3% and 91.4%, negative predictive value was 53.1%, 64.3% and 74.1% and accuracy was 66.1%, 75.8% and 83.9%, respectively, at visits one, two and three. Thus, with increasing visits, an increase in all the components of diagnostic efficacy was observed with maximum efficacy achieved at the third visit and minimum at the first visit. These observations coincide with the conceptual basis provided by Gudmundsson et al. (1988) [22], however, Dhand et al. (2011) [23] reported the umbilical artery PI to be only 44% and 61.5% sensitive and specific in the prediction of IUGR. Compared to this, we obtained a better performance for all the parameters. One of the reasons for this could be the fact that while Dhand et al. (2011) [23] carried out their study as a case-control study, in the present study, we had only a prospective case series of high IUGR risk-designated pregnancies. The variability in diagnostic efficacy of umbilical artery PI is reported to vary in different studies, and mostly it is because of the difference in the design or method of estimation, for example, Narula et al. (2009) [24] reported a sensitivity of 94% for combined indices of the umbilical artery.

Umbilical artery PI is relatively a more-specific criterion than a sensitive criterion. In the present study though, the shift in sensitivity was from 61.5% at the first visit to 82.1% at the third visit and the shift in specificity was from 73.9% to 87.0%. In fact, by the third visit, diagnostic efficacy, in general, increased for all the components viz. sensitivity, specificity, positive predictive value (PPV) and negative predictive value (NPV). In literature, it has been reported that pulsatility index decreases initially and then increases; thus, by the third visit (late third trimester), the pulsatility index is at its peak and, hence, the criteria become more sensitive. In cases with placental insufficiency, the diastolic flow decreases and thus results in a higher PI value [22].

In the present study, umbilical artery RI was also seen to have a good discriminant value but with relatively lower sensitivity as compared to PI. In different studies too, umbilical artery RI has been reported to have low sensitivity and high specificity. Compared to this, Lakhkar et al. (2006) [10] reported that umbilical artery RI had a sensitivity of 58%, specificity of 71.7%, PPV of 35% and NPV of 86.8%, thereby showing a diagnostic accuracy of 56.8% for a major adverse outcome in clinically suspect IUGR cases and a sensitivity, specificity, PPV and NPV of 44.4%, 81.8%, 80% and 47.3%, respectively ,for minor adverse outcomes. Aali et al. (2010) [25] evaluated the efficacy of umbilical artery RI for the evaluation of pregnancy complications, such as preeclampsia, and found that at a cut-off value of 0.64, it was 100% sensitive but only 44% specific; however, at a higher cut-off of 0.81, it was only 28% sensitive and 100% specific. In the present study, owing to clinically suspect and grayscale confirmed IUGR cases, both sensitivity and specificity were fair. However, the lower negative predictive value could be explained easily on the basis of the higher prevalence of IUGR in the present study.

For the umbilical artery S/D ratio, the present study showed a low discriminant value for IUGR prediction. Similar to the present study, Chanprapah et al. (2004) [21] also observed a lower discriminant value of umbilical artery S/D ratio for the prediction of IUGR. However, in another study by Wang et al. (1996) [26], the sensitivity, specificity and PPV of the umbilical artery S/D ratio was observed to be 80.0%, 83.7% and 50.0% at 24-30 weeks gestation time, which is much higher than that observed in the present study. However, whether this difference is dependent on incidence cannot be ruled out, as their study had only 16.9% IUGR cases. The low positive predictive value in their study indicates the selection of relaxed criteria for identification. As there were more negative cases as compared to positive cases in their study, the loss of specificity was not too high. In the present study, we have observed a sensitivity of 53.8% and a negative predictive value of 51.4% for patients at the first visit, which increased only slightly by the third visit to reach 61.5% and 57.1%, respectively. The results at a late gestational age, as obtained in the present study, are similar to those obtained by Kofnias et al. (1990) [27] who observed a sensitivity, specificity, PPV and NPV of 71%, 93%, 83% and 90%, respectively, in a study with an incidence of 30.9% IUGR. It would be pertinent to mention here that the study of Kofnias et al. had included subjects in their third trimester. Thus, it seems that with the progression of pregnancy, the umbilical artery S/D ratio becomes a useful tool to predict IUGR.

In the present study, we found MCA PI and RI values to be more specific than sensitive. These findings are in consonance with the findings of Bano et al. (2010) [4] who while using the same cut-off observed the sensitivity, specificity, PPV and NPV of MCA PI to be 8.9%, 100%, 100% and 52.3%, respectively, with a diagnostic accuracy of 54.4%. While studying the usefulness of MCA parameters, we observed an increasing trend of sensitivity for MCA RI with increasing gestational age. This might be because of the dynamic behavior of MCA RI as reported by Kurmanavicius et al. (1997) [13]. They observed a parabolic pattern of change in MCA RI with increasing age; this implies that a single cut-off value should not be employed for the prediction of IUGR. In fact, in their article, they had given a regression equation dependent on gestational age to calculate the appropriate cut-off for different gestational ages. In the present study, we have used a single cut-off criterion and found results that did not have much predictive value.

In the present study, the third trimester MCA/UI ratio for PI was found to be 82.1% sensitive and 95.7% specific with a PPV, NPV and diagnostic accuracy of 97%, 75.9% and 87.1% only. However, the MCA/UI ratio for RI was found to be only 71.8% sensitive and 82.6% specific with a PPV, NPV and diagnostic accuracy of 87.5%, 63.3% and 75.8%. In the present study, the MCA/UI ratio showed to be 76.9% sensitive, 78.3% specific and with positive predictive, negative predictive and accuracy values of 85.7%, 66.7% and 77.4%, respectively.

In a previous study by Bano et al. (2010) [4], the MCA/UI PI ratio was observed to be 83.3% sensitive and 100% specific with a PPV, NPV and diagnostic accuracy of 100%, 94.3% and 95.6%. Fong et al. (1999) [28] reported the efficacy of the UI/MCA PI ratio in the prediction of any adverse perinatal outcome to be 51.3% sensitive and 80.6% specific with a PPV and NPV of 48.1% and 82.5%, while for the prediction of only major adverse outcomes, the sensitivity and specificity was 62.5% and 75.5% with a PPV and NPV of 18.5% and 95.8% only. It can be seen that with the decreasing incidence, the positive predictive value decreases while the negative predictive value increases. In the present study, where the incidence of IUGR was 70%, we had high sensitivity as well as specificity. Our findings are close to the findings of Bano et al. [4], who too carried out their study in a high incidence situation like ours. As regards MCA/UA RI, the findings in the present study are better than the results of El-Sokkary et al. (2011) [29] who reported it to be 47%, sensitive, 90% specific with a 95% positive predictive and a 43% negative predictive value.

In the present study, ductus venosus SIA values at the first visit, the sensitivity, specificity, positive predictive, negative predictive and accuracy values were 25.6%, 82.6%, 71.4%, 39.6% and 46.8%, respectively. At the second visit, the corresponding values were 28.2%, 87.0%, 78.6%, 41.7% and 50%, respectively, and corresponding third visit values were 41.0%, 91.3%, 88.9%, 47.7% and 59.7%, respectively. Although an increase in accuracy was observed to the tune of ~13% between the first and third visits; however, at all the visits, the sensitivity was too low to be of clinical use. The findings in the present study do not correspond with the observations made by Picconi et al. (2008) [19], who found ductus venosus SIA values to be 100% sensitive and 100% specific for the prediction of live births and 67% sensitive and 94% specific for neonatal death. It was surprising that they took two different cut-off values to predict the outcome of a similar nature. In the present study, we took both the criteria as indicators of IUGR; however, it did not turn out to be of much clinical use.

In the present study, the first visit, second visit and third visit sensitivity, specificity, positive predictive, negative predictive and accuracy values for vertebral artery RI were 43.6%, 52.2%, 60.7%, 35.3% and 46.8%; 56.4%, 60.9%, 71.0%, 45.2% and 58.1%; and 0.8%, 73.9%, 82.4%, 60.7% and 72.6%, respectively. Overall sensitivity, specificity, positive predictive, negative predictive and accuracy values were 57.3%, 62.3%, 72.0%, 46.2% and 59.1%, respectively. Morales-Roselló and Peralta-Llorens (2012) [30] were of the view that vertebral artery RI might have a potential to identify the group of fetuses with brain sparing and severe IUGR. However, in their assessment, they reported a variable performance of VA RI, depending on variable criteria for IUGR definition.

## Conclusions

The findings in the present study thus suggest that Doppler flowmetry is a useful method for the prediction of IUGR in high-risk pregnancies. Among different markers examined in the study, umbilical artery PI had high efficacy in both early as well as the latter part of the third trimester. However, third visit C/U PI had high sensitivity and specificity. The findings of the present study must be viewed in context with a high-risk population with clinical suspicion of IUGR. Further cross-sectional studies on a larger population are recommended for pregnant women with different risk levels.
